# Dynamic Mechano-Regulation of Myoblast Cells on Supramolecular Hydrogels Cross-Linked by Reversible Host-Guest Interactions

**DOI:** 10.1038/s41598-017-07934-x

**Published:** 2017-08-09

**Authors:** Marcel Hörning, Masaki Nakahata, Philipp Linke, Akihisa Yamamoto, Mariam Veschgini, Stefan Kaufmann, Yoshinori Takashima, Akira Harada, Motomu Tanaka

**Affiliations:** 1 Institute for Integrated Cell-Material Science (WPI iCeMS), Kyoto University, Kyoto, 606-8501 Japan; 2 Project Research Center for Fundamental Sciences, Graduate School of Science, Osaka University, 1-1 Machikaneyama-cho, Toyonaka Osaka, 560-0043 Japan; 3 Physical Chemistry of Biosystems, University of Heidelberg, D69120 Heidelberg, Germany; 4 Department of Macromolecular Science, Graduate School of Science, Osaka University, 1-1 Machikaneyama-cho, Toyonaka Osaka, 560-0043 Japan; 5 Present Address: Institute of Biomaterials and Biomolecular Systems (IBBS), University of Stuttgart, 70569 Stuttgart, Germany; 6 Present Address: Division of Chemical Engineering, Department of Materials Engineering Science, Graduate School of Engineering Science, Osaka University, 1-3 Machikaneyama-cho, Toyonaka Osaka, 560-8531 Japan

## Abstract

A new class of supramolecular hydrogels, cross-linked by host-guest interactions between β-cyclodextrin (βCD) and adamantane, were designed for the dynamic regulation of cell-substrate interactions. The initial substrate elasticity can be optimized by selecting the molar fraction of host- and guest monomers for the target cells. Moreover, owing to the reversible nature of host-guest interactions, the magnitude of softening and stiffening of the substrate can be modulated by varying the concentrations of free, competing host molecules (βCD) in solutions. By changing the substrate elasticity at a desired time point, it is possible to switch the micromechanical environments of cells. We demonstrated that the Young**’**s modulus of our **“**host-guest gels**”**, 4–11 kPa, lies in an optimal range not only for static (*ex situ*) but also for dynamic (*in situ*) regulation of cell morphology and cytoskeletal ordering of myoblasts. Compared to other stimulus-responsive materials that can either change the elasticity only in one direction or rely on less biocompatible stimuli such as UV light and temperature change, our supramolecular hydrogel enables to reversibly apply mechanical cues to various cell types *in vitro* without interfering cell viability.

## Introduction

Ample experimental evidence suggested that biological cells do not only sense and respond to biochemical cues from the surrounding environment but also actively react to the mechanical properties (elasticity) of the extracellular microenvironments^1,2^. There are two signaling pathways regulating such mechano-sensing machineries. First, the clusters of integrin receptors, called focal adhesions, trigger the downstream cascades of intracellular signaling pathways, called “outside-in” signaling. This leads to mechanical force generation via contraction of actin-myosin (actomyosin) complexes^3^. The resistance of the extracellular matrix (ECM) against the traction force determines the connection between integrin clusters and actomyosin complexes, mediated by talin and vinculin. Second, the mechanical stimulation of actomyosin complexes by external cues can also cause the conformational change in the cytoplasmic domains of integrin and strengthen the binding to the extracelluar matrix, called as “inside-out” signaling^4^.

To date, various ECM models based on chemically cross-linked hydrogels have been designed^5^ to investigate the regulatory principles of mechano-sensing. Through fine adjustment of cross-linker concentrations and the reaction time^6,7^, one can control the bulk elasticity (Young’s modulus) of a given gel substrate “*ex situ*”. Such materials have been used to gain insight into the vital role of elasticity compliance between cells and ECM in optimizing the cell morphology^8–10^, regulating the migratory behavior^11,12^, and controlling the stem cell differentiation^13,14^. On the other hand, many *in vivo* studies and experiments using organotypic cultures demonstrated that dynamic changes in the ECM stiffness influence various key functions of cells. For example, transplanted mesenchymal stem cells exhibit a remarkable enhancement of bone regeneration upon the degradation of soft alginate matrix^15^. It was also found that mesenchymal stem cells transplanted near the liver tissue did not adhere or repair the damaged tissue, but settled down in the periportal space^16^. Another medically relevant example is a clear correlation between the ECM density and the migration pattern of cancer cells^17^. These findings inspired the design of a new ECM model, whose mechanical properties can be altered in a time-dependent manner^18^.

Recent studies in the field of supramolecular chemistry have shown that reversible bonds formed through specific intermolecular interactions can impart dynamic and adoptive properties to polymer materials^19,20^. To date, a number of polymer materials that respond to external stimuli such as light, redox reaction, pH level, heat, and electric/magnetic fields, have been designed to regulate their mechanical properties^21,22^. However, the external stimuli used in many cases, such as UV light^23^ and temperature change^24,25^, are cytotoxic and interfere with the cell viability. Several recent studies demonstrated that the thiolated hyaluronic acid^26^ and gelatin^27^ form chemical gels via disulfide bonds, whose Young’s modulus decreased by adding dithiothreitol. However, such materials have a fundamental drawback: the change in the substrate stiffness by the cleavage of disulfide bonds goes only in one direction. Once a bond is cleaved, it can hardly be recovered. Polymers increasing the density of covalent cross-links by external stimuli share the same problem. Once a covalent bond is formed, it is very hard to cleave it.

Previously, we proposed the use of physically cross-linked hydrogels composed of an inter-connected micellar network of triblock copolymer chains possessing pH-responsive blocks at two ends. The elasticity of pH-responsive gels can be modulated by pH titration. The morphological dynamics and adhesion strength of several different cell types could be achieved^28–30^. However, although we observed no major interference with cell viability, a new class of hydrogel substrates that can reversibly alter the elasticity at pH 7.4 is more desirable for broader applications. In this study, we selected cyclodextrin (CD) as a milder chemical stimulus, which is a cyclic oligosaccharide that can act as the “host” for different hydrophobic “guest” molecules. Harada *et al*. have utilized host-guest interactions of CD, and designed a rich variety of hydrogels that are cross-linked with the host-guest interactions^31,32^. Since the association and dissociation of host-guest pairs can readily be achieved by adding either host or guest molecules in solutions, the host-guest polymer materials allow a number of applications^33,34^, such as stimuli-responsive actuators^35^ and self-healing materials^31,36^.

We functionalized the side chain of acrylamide monomers with β-cyclodextrin (βCD), and used them as the host. As the guest moiety, we selected adamantane, which fits well into the hollow cavity inside βCD. By optimizing the mixing ratio between pure acrylamide monomers (matrix) and acrylamide monomers modified with host/guest moieties, we fabricated hydrogels that possess the elastic modulus suited for the culture of myoblasts. This substrate material is advantageous over other commonly used hydrogels: First, the poylacrylamide backbones have widely been utilized for the culture of various cell types. Second, both βCD and adamantane are known to be non-cytotoxic^37,38^. This enabled us to use free cyclodextrin molecules as a molecular stimulus (competitor) that can fine-adjust the chemical equilibrium of cross-links and thus can reversibly alter the substrate elasticity without damaging cells. Details of the experimental findings are summarized in the following sections.

## Materials and Methods

### Materials

Acrylamide (AAm), CDCl_3_, and D_2_O were purchased from Wako Pure Chemical Industries, Ltd. βCD was obtained from Junsei Chemical Co., Ltd. Triethylamine (Et_3_N), sodium hydroxide, hydrochloric acid, ammonium peroxodisulfate (APS), *N*,*N*,*N*′,*N*′–tetramethylethylenediamine (TEMED), and *N*,*N*′–methylenebis(acrylamide) (MBAAm) were purchased from Nacalai Tesque Inc. Acryloyl chloride and vinyltrimethoxysilane were purchased from Tokyo Chemical Industry Co., Ltd. DMSO–*d*_6_ was obtained from Merck & Co., Inc. RPMI-1640 medium, fetal bovine serum, penicillin and streptomycin were purchased from Sigma-Aldrich, Co. LifeAct-TagGFP2 and the Torpedo® lipofection reagent were purchased from ibidi GmbH. A highly porous synthetic resin (DIAION HP–20) used for column chromatography was purchased from Mitsubishi Chemical Co., Ltd. Water used for the preparation of the aqueous solutions was purified with a Millipore Integral MT system. Other reagents were used without further purification.

### Sample preparation

Round cover glass (∅ = 25 mm) were cleaned using a modified RCA method^39^. The substrates were immersed in a 5% (v/v) solution of vinyltrimethoxysilane in toluene and shaken for 18 h at room temperature. After the sequential rinsing in acetone, ethanol, and deionized water, the glass substrates were baked at 140 °C for 1 h in air. The vinyl-silanized glass substrates were protected from UV irradiation until final use.

βCD—Ad gel (*x*,*x*) was prepared according to previous literatures^32,40^. In brief: 6–acrylamido–βCD (βCD—AAm, *x* mol%) and adamantane-acrylamide (Ad—AAm, *x* mol%) were mixed in water at 90 °C in a silicone oil bath for 3–4 h. After cooling, AAm (100–2*x* mol%) was added. Here, *x* represents mol% of βCD–AAm and Ad–AAm monomers. *C*_m_ represents the total concentration of βCD—AAm, Ad—AAm, and AAm. After dissolving all monomers, APS and TEMED were added, and a 50 μL portion of the mixed solution was injected in a gap between a vinyl-silanized glass substrate and a hydrophilic, plasma-treated cover glass and kept for 15 min. After removing the cover glass, the sample was successively soaked in excess of DMSO/water (1:1, v/v) for 24 h and in water for 48 h to remove the residual chemicals. The surface of hydrogel substrates was functionalized with fibronectin via bifunctional cross-linker Sulfo-SANPAH (Thermo Scientific), following previous publications (Supporting Information S1)^41,42^.

### Mechanical properties of host-guest gels

The bulk elasticity of host-guest gels was measured by a Rheoner RE–33005 creep meter (Yamaden Ltd., Tokyo). The elasticity of host-guest gels near the surface was determined by nano-indentation with an atomic force microscope (NanoWizard, JPK Instruments, Berlin). As reported previously, the density of hydrated polymers near the surface is diffusive and thus cannot be treated like a clear boundary with a Gaussian roughness^43–45^. In order to avoid the artifacts caused by indenting diffusive interface that has a larger length scale than the curvature radius of cantilevers (typically 20–30 nm), we indented the sample with a Pyrex-nitride spherical colloidal probe (*R* = 5 μm) attached to a silicon-nitride cantilever with a nominal spring constant of 0.08 N/m (CP-PNP-BSG; Olympus Optical). The Young’s modulus *E* of the gel was calculated from the nonlinear least-square fitting of the force-indentation curves^46,47^:

1 $$F=4E{R}^{1/2}\cdot {[3{(1-\nu )}^{2}]}^{-1}\cdot {\delta }^{3/2}$$

where *F* is the force applied to the indenter, *ν* = 0.5 the Poisson’s ratio, and *δ* the indentation depth^48^.

Unless stated otherwise, all the data points are from more than three independent measurements, and the error bars in each figure correspond to the standard deviations.

### Cell culture

Mouse myoblast cells (C2C12, <20 passages, RIKEN BRC Cell Bank) were cultured in RPMI-1640 medium supplemented with 10 wt% of fetal bovine serum, 100 U/mL penicillin and 100 μg/mL streptomycin. The cells were detached from the culture-flasks by enzymatic digestion using trypsin-EDTA (0.25%, Sigma), and 1 × 10^4^ cells were seeded on the fibronectin-coated substrate 24 h before the observation. The actin filaments in live cells were visualized by transfecting C2C12 with LifeAct-GFP following the manufacturer’s protocol (ibidi GmbH). Prior to the experiments, we confirmed that free βCD molecules in solutions have no cytotoxicity by the WST assay (Supporting Information S2).

### Morphology analysis

Cell dynamics on hydrogels were monitored by an inverted fluorescence microscope (Zeiss) and a Fast-Scan Confocal Fluorescence Microscope (Nikon, A1R). Morphological parameters, such as projected area *A*, aspect ratio *AR* (the ratio between major and minor axes), and circularity

2 $$C=4\pi A/{(perimeter)}^{2}$$

Of the adherent C2C12 cells were calculated using algorithms written in Matlab (Mathworks). The nematic order parameter of actin cytoskeletons <*S*> is calculated from the actin filaments as described before^29,49,50^. The original image was convoluted with the *n* eLoG kernels and the maximum response image was calculated for each pixel, as

3 $${I}_{{\rm{\max }}}(x,y)=\,\max \,[eLoG(n)\times I(x,y)]$$

*I*_max_ was processed by an intensity-threshold to obtain the image of the segmented stress fibers^51^, and the fibers with the same rotational direction with less than 10 pixels were removed. The order parameter

4 $$\langle S\rangle =\langle \,\cos \,2\theta \rangle $$

was calculated from the histogram of fiber orientations that were scaled by the corresponding fluorescence intensities to account for the local concentration of actin filaments.

### Data availability

All data generated or analyzed during this study are included in this published article (and its Supplementary Information files).

## Results and Discussion

**Figure 1:**
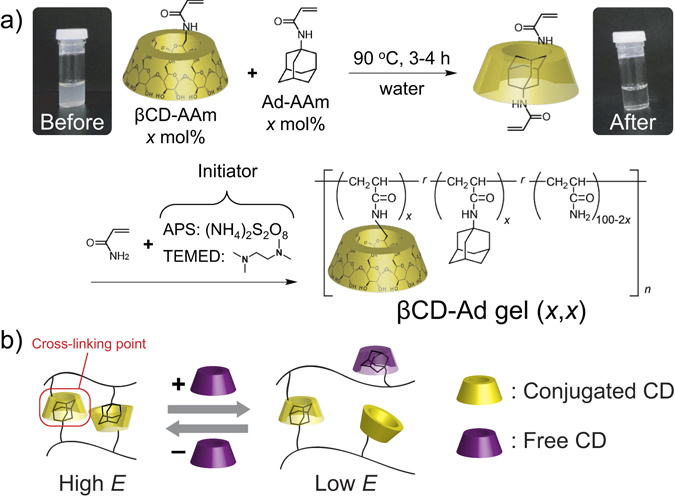
Working principle of host-guest gel. (**a**) Preparation and chemical structure of βCD–Ad gel (*x*,*x*). *x* represents the mol% contents of βCD and Ad units. (**b**) Reversible switching of βCD–Ad bonds in response to free βCD molecules (purple) in solution. Under equilibrium, the fraction of βCD–Ad bonds connected to the polymer chains depends on βCD concentration, which allows for the dynamic modulation of Young’s modulus *E*.

Figure 1a shows preparation and chemical structure of the host-guest gel (βCD–Ad gel (*x*,*x*)) used as cell culture substrates in this study. The gel was prepared by radical polymerization after dissolving the hydrophobic guest monomers (Ad–AAm) by forming the inclusion complex with the host monomer (βCD–AAm). These monomers were terpolymerized with AAm in aqueous solution, forming a hydrogel. *x* represents mol% of βCD–AAm and Ad–AAm monomers in the monomer solution. Figure 1b shows how the fraction of βCD–Ad cross-linkages can be modulated by the presence of free βCD molecules in aqueous solution. Free βCD molecules act as competitors and reduce the number of βCD side chains conjugated with Ad. As a consequence, one can fine-adjust the degree of cross-linking and thus the Young’s modulus *E* of gels by adjusting βCD concentration under equilibrium. It is notable that the gel can recover the original elasticity value once immersed into βCD-free solution.

**Figure 2:**
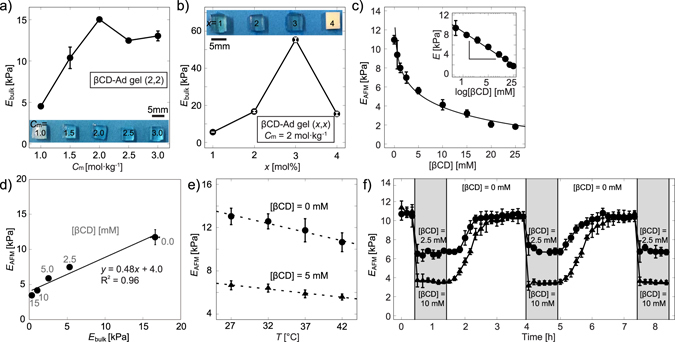
Macroscopic and microscopic gel assessment. (**a**) Young’s modulus *E*_bulk_ of βCD–Ad gel (2,2) vs. total monomer concentration *C*_m_ = 1.0, 1.5, 2.0, 2.5, and 3.0 mol kg^−1^, measured by a creep meter. (**b**) *E*_bulk_ plotted as a function of the mol% content of βCD–AAm and Ad–AAm monomers in the monomer solution (*x*), while keeping *C*_m_ = 2.0 mol·kg^−1^ constant. (**c**) *E*_AFM_ of βCD–Ad gel (2, 2) vs. βCD concentration, measured at RT. The change in elasticity shows a clear dependence on βCD concentration; ${\rm{\Delta }}{E}_{AFM}\propto \,\mathrm{log}[\beta CD]$. (**d**) Correlation between *E*_bulk_ measured by a creep meter and *E*_AFM_ measured by AFM indentation. βCD–Ad gel (2,2) with *C*_m_ = 2.0 mol kg^−1^ was immersed into RPMI 1640 medium containing 0, 2.5, 5.0, 10, and 15 mM of βCD. (**e**) Temperature dependence of *E*_AFM_ in the presence and absence of 5 mM βCD. (**f**) *In situ* modulation of *E*_AFM_ at *T* = 37 °C. Reversible switching of the substrate elasticity was confirmed by alternatively changing βCD concentration in the solution between 0 and 2.5 mM (●) as well as 0 and 10 mM (▲).

Figure 2a represents the influence of total concentration of monomers *C*_m_ on the optical transparency and the elastic modulus obtained by the compression test with a creep meter (*E*_bulk_) at a constant fraction of host- and guest monomers (*x* = 2 mol%). As presented in the figure, the sample seemed opaque at a low monomer concentration, *C*_m_ = 1.0 mol kg^−1^. The increase in *C*_m_ to 2.0 mol kg^−1^ led to a monotonic increase in the elastic modulus to *E*_bulk_ = 15 kPa, and the gel became transparent at *C*_m_ ≥ 1.5 mol kg^−1^. Further increase in *C*_m_ did not result in any remarkable change in *E*_bulk_. Previously, Horkay *et al*. reported that the saturation of *E*_bulk_ occurs at a volume fraction (*φ*) of *φ* ≈ 0.2 in the case of poly(acrylamide)^52^. In our experimental system, *C*_m_ > 2.0 mol kg^−1^ corresponds to *φ* > 0.2. Thus, we concluded *C*_m_ = 2.0 mol kg^−1^ is optimal for our purpose. Figure 2b shows the influence of the molar fraction of host- and guest monomers *x* on *E*_bulk_, while keeping *C*_m_ constant at 2.0 mol kg^−1^. A clear increase in *E*_bulk_ in accord with the increase in *x* from 1 to 3 mol%. However, we found that the gel becomes opaque and *E*_bulk_ took a much lower value at a high molar fraction, *x* > 3 mol%. Based on these two experimental findings, we kept *C*_m_ = 2.0 mol kg^−1^ and *x* = 2 mol% in the following.

To examine if one can modulate the elasticity of host-guest gels by adding host molecules, we measured the Young’s modulus of hydrogel samples by two methods: *E*_bulk_ was obtained from the linear regime of macroscopic stress-strain curves, and *E*_AFM_ was determined by AFM nano-indentation. As presented in Fig. 2c, *E*_AFM_ exhibited a remarkable decrease by increasing βCD concentration, following the change in chemical potential of βCD,

5 $${E}_{AFM}\propto \,\mathrm{log}[\beta CD]$$

Figure 2d represents the plot of *E*_bulk_ vs. *E*_AFM_ measured at different βCD concentrations. It should be noted that the *E*_bulk_ value remained stable over 24 h when a hydrogel substrate was immersed in the medium with a defined βCD concentration, confirming that *E*_bulk_ was measured under equilibrium. Excellent agreement between *E*_bulk_ and *E*_AFM_ implies that free βCD molecules in solution can diffuse inside the hydrogel samples, resulting in the modulation of the Young’s modulus of the whole hydrogel substrates with the thickness up to 200 µm. In the next step, *E*_AFM_ was measured in the absence and presence of 5 mM βCD under different temperature conditions (Fig. 2e). The elastic modulus exhibited a clear decrease both in the presence and absence of free βCD in solution at elevated temperature. This finding suggests that the gels consist of three-dimensional networks of high molecular weight polymers like rubber and thus the elasticity is dominated by entropy^53^. Figure 2f represents the dynamic modulation of *E*_AFM_ in response to the repetitive exchange of media with and without free βCD in solutions at *T* = 37 °C. Initially, the elasticity of hydrogel substrates in the absence of free βCD in solutions ([βCD] = 0 mM) was *E*_AFM_ ≈ 11 kPa, and the exposure to the medium containing 2.5 mM free βCD in the solution (●) led to an abrupt decrease in the elasticity, *E*_AFM_ ≈ 7 kPa. After confirming the equilibration, the medium was exchanged to βCD-free medium, which resulted in a recovery of the elasticity to the initial level, *E*_AFM_ ≈ 11 kPa. Several cycles were repeated to guarantee that the modulation of the substrate elasticity is fully reversible. The experiments with 10 mM βCD (▲) also exhibited a fully reversible modulation of *E*_AFM_. As expected from the results presented in Fig. 2d, we obtained a lower *E*_AFM_ ≈ 4 kPa with 10 mM βCD, demonstrating that the magnitude of switching can be adjusted by βCD concentration.

**Figure 3:**
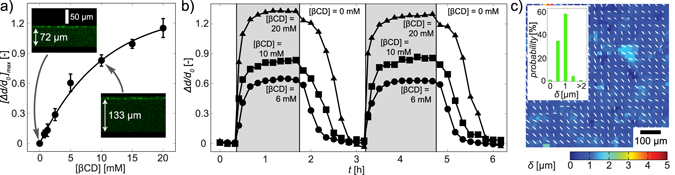
Reversible switching of thickness by medium exchange. (**a**) Maximum swelling ratio [Δ*d*/*d*_0_]_max_ plotted as a function of βCD concentration. The insets show two representative cross-sectional images of gels at [βCD] = 0 and 10 mM, reconstructed from the confocal fluorescence microscopy images. Here, fibronectin labeled with HiLyte Fluor™ 488 was used to confirm the uniform functionalization of gel surfaces. The black line is the fit of the equation $f([{\rm{\beta }}\mathrm{CD}])\propto 1-\exp (-[{\rm{\beta }}\mathrm{CD}]/{[{\rm{\beta }}\mathrm{CD}]}_{c})$ (6) with the characteristic concentration [βCD]_c_ = 10.3 mM. (**b**) Reversible switching of Δ*d*/*d*_0_ by repetitive exchanges of media with different βCD concentration; 6 mM (●), 10 mM (■), and 20 mM (▲). (**c**) The lateral displacement of fluorescent beads embedded in the gel caused by the incubation with 10 mM βCD for 40 min. The magnitude of displacement is indicated by the color code, while the direction is indicated by the arrows. The histogram in the inset represents the distribution of displacements, implying that the lateral displacement is ≤1 µm for more than 90% of the beads.

Figure 3a represents the maximum swelling ratio [Δ*d*/*d*_0_]_max_ plotted as a function of βCD concentration. The acquired data can be represented empirically by a single negative exponential fit (Fig. 3a, solid line) with the characteristic concentration [βCD]_c_ = 10.3 mM. The addition of 10 mM βCD led to an increase in the film thickness from 72 µm to 133 µm, resulting in the swelling ratio of about 0.9. The insets show two confocal images of the gel (side views), whose surfaces were functionalized with the fluorescently labeled fibronectin. Figure 3b shows the dynamic change in the swelling ratio (∆*d*/*d*_0_) as a function of time under repetitive exchanges of the βCD-free medium and the media with [βCD] = 6 mM (●), 10 mM (■), and 20 mM (▲). The swelling ratio reaches to its saturation level in about 1 h after the medium exchange in both directions. The apparent difference between the change in *E*_AFM_ and the change in swelling ratio can be attributed to the difference in the length scales between the AFM indentation (<1 μm) and ∆*d* (>50 μm) as reported previously^54^.

Biological cells do not only deform the substrate by generating active traction forces^55–57^ but also react to the lateral strain of substrates^58,59^. Therefore, towards the dynamic regulation of cells, it is highly important to check if the swelling of hydrogels is isotropic. For this purpose, we embedded fluorescently labeled latex particles in the host-guest gel, and monitored the lateral displacement caused by swelling using confocal microscopy (Supporting Information S4). Figure 3c represents the lateral deformation of the gel analyzed by measuring the displacement of embedded fluorescent beads and using particle image velocimetry (PIV) after the incubation in the medium containing 10 mM βCD. The magnitude of displacement is indicated by the color code, while arrows indicate the direction. Although the swelling of the film is Δ*d*_max_ ≈ 60 µm, as shown in Fig. 3a, the histogram of displacement (inset) implies that the lateral displacement is ≤1 µm for more than 90% of the beads. This finding confirms that exposure to βCD induces swelling of host-guest gels in the direction normal to the substrate but does not exert shear force on the cells caused by lateral deformation. As reported previously^60^, such an anisotropic swelling of hydrogels could be attributed to the physical constrain of the host-guest gel, whose bottom side is covalently anchored to the solid substrates.

**Figure 4:**
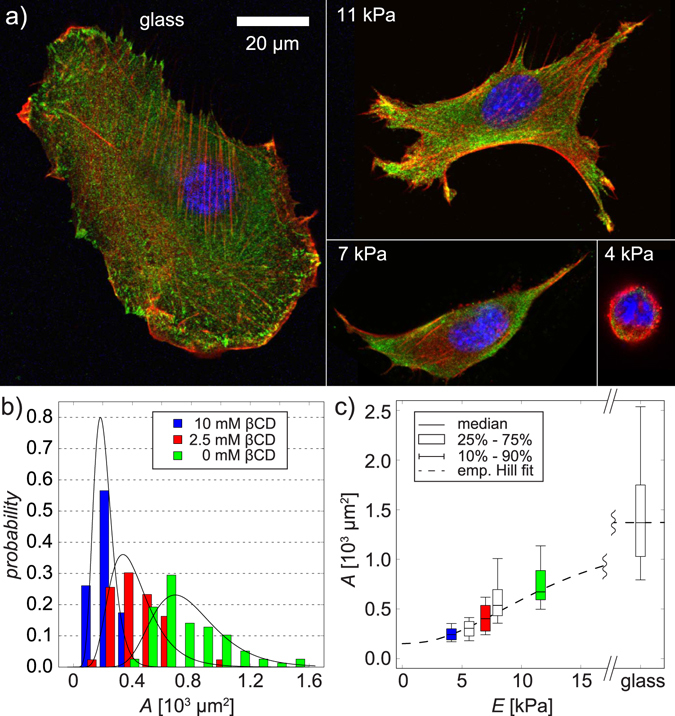
*Ex situ* mechano-sensing of C2C12. (**a**) Confocal fluorescence images of C2C12 cells on glass substrates (*E* ≈ 50 GPa, control), and host-guest gel substrates with *E* = 11, 7, and 4 kPa. All the substrates were functionalized with fibronectin. Vinculin (green), actin (red), and nuclei (blue). (**b**) Histograms of the projected areas *A* of C2C12 cells at [βCD] = 0 (green), 2.5 (red), and 10 mM (blue), taken from a total number of *n* > 150 cells. The distribution for each condition could be well fitted with a log-normal function. (**c**) Projected area as a function of the elastic modulus of the gel, fitted with the empirical Hill equation (broken line).

As the first step towards the mechanical regulation of cells using host-guest gel substrates, we monitored how C2C12 cells sense the substrate elasticity *ex situ*. Here, we seeded C2C12 myoblasts on four different substrates; glass (*E* ≈ 50 GPa, control) and host-guest gel substrates equilibrated with [βCD] = 0 (*E* ≈ 11 kPa), 2.5 (*E* ≈ 7 kPa), and 10 mM (*E* ≈ 4 kPa). Figure 4a shows the representative confocal fluorescence images of C2C12 cells on four substrates, showing the overlay of vinculin (green), actin (red), and nuclei (blue). First, according to the increase in the substrate elasticity *E*, we found more concentrated focal adhesions and more pronounced actin stress fibers. Moreover, we found that the projected area of cells *A* monotonically increased according to the increase in *E*. The statistical reliability of the observed tendency was verified by taking the histogram from more than 23 cells for each substrate (Fig. 4b), which can be well fitted with a log-normal function. As presented in Fig. 4c, the dependence of projected area *A* on substrate elasticity *E* can be fitted by the empirical Hill equation (broken line):

7 $$A(E)=\frac{{A}_{max}}{({(\frac{{E}_{\frac{1}{2}}}{E})}^{m}+1)}+{A}_{min}.$$

The median area on the glass was taken as *A*_max_ = 1217 μm^2^, while the median area on the softest substrate (*E* ≈ 4 kPa) was set as the minimum level *A*_min = _150 μm^2^. The half level, *E*_1/2_ ≈ 13 kPa, represents the characteristic *E* value for the cell spreading, while *m*_ = _2.2 is the cooperativity coefficient^61^. The obtained *E*_1/2_ value for C2C12 on host-guest gels seems to agree well with those of C2C12 on other materials reported previously, such as *E*_1/2_ ≈ 23 kPa on pH sensitive gels^28^ and *E*_1/2_ ≈ 7.6 kPa on photo-crosslinked gelatin gels^50^. Actually, *E*_1/2_ ≈ 13 kPa agrees very well with the optimal elastic modulus of *E* ≈ 12 kPa for actomyosin striations^9^ as well as for the stabilization of cardiac conduction^62,63^. Therefore, our host-guest gels are ideally suited to modulate the Young’s modulus of the substrates between the optimal level and the softer regime for myoblasts.

**Figure 5:**
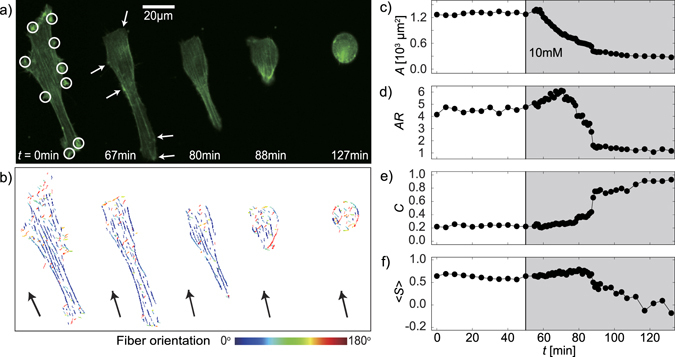
*In situ* mechano-response of C2C12. Dynamic response of C2C12 myoblasts upon the softening of βCD–Ad gel (2,2) from *E* ≈ 11 to 4 kPa, caused by the exchange to the medium containing 10 mM of βCD. (**a**) Snapshot images of a C2C12 cell from the live cell imaging (LifeAct-GFP). (**b**) Actin cytoskeletons extracted from the images. Changes in (**c**) projected cell area *A*, (**d**) aspect ratio *AR*, (**e**) circularity *C*, and (**f**) nematic order parameter of actin cytoskeleton <*S>* are plotted over time. The medium was exchanged at *t* = 50 min, and medium containing βCD was continuously supplied in the shaded time zone.

In the next step, we focused on the dynamic, *in situ* response of C2C12 to the softening of the substrate elasticity from *E* ≈ 11 kPa to 4 kPa by the transient transfection with LifeAct-GFP to visualize actin cytoskeletons. In Fig. 5a, several representative snapshot images selected from the live cell imaging were presented. Initially, long actin cytoskeletons form bundles (stress fibers) near the cell periphery to maintain the tension on the substrate with *E* ≈ 11 kPa (“contractile” state). Upon the exchange from βCD-free medium to medium containing 10 mM of βCD at *t* = 50 min, the cell started changing its shape after some lag time (∆*t* ≈ 10 min). As a result, the area *A* reached to the steady state at *t* ≈ 90 min, where the cell took a round shape (“resting” state). As shown in Fig. 5b, the actin cytoskeletons became shorter and more isotropic during the transition from the “contractile” to “resting” states. To gain further insights into the dynamic response, changes in the standard morphometric parameters were plotted as a function of time; projected area *A* (Fig. 5c), aspect ratio *AR* (Fig. 5d), and circularity *C* (Fig. 5e). The projected area *A* starts monotonical decreasing after *t* ≈ 60 min, which coincides with the first detachment of focal adhesions. The fact that *A* did not show a remarkable increase seems reasonable from the lack of lateral expansion by the addition of competitive βCD (Fig. 3c). This time lag ∆*t* is slightly different between cells because of the stochasticity of the bond detachment under the softening of the substrates. On the other hand, the aspect ratio *AR* first exhibited an increase until *t* ≈ 70 min, followed by an abrupt decrease. The apparent increase in *AR* can be attributed to the strong pinning of the focal adhesions (indicated by white circles in Fig. 5a), which remained even after the detachment of some other focal contacts (indicated by white arrows in Fig. 5a). Nevertheless, *AR* rapidly decreased once these contacts were detached, reaching to *AR* ≈ 1 (round, resting cell). The circularity *C* remained almost constant at a low level (*C* ≈ 0.2) until *AR* started dropping rapidly. This tendency seems consistent with the fact that C2C12 did not exhibit remarkable protrusions (filipodia) even at the contractile state. The identification of actin filaments from the live cell images (Fig. 5b) further allows for the calculation of nematic order parameter of actin filaments, $\langle S\rangle =\langle \,\cos \,2\theta \rangle$ (4), where *θ* is defined as an azimuth angle between the actin filament and the major axis of the cell. As presented in Fig. 5f, the nematic order parameter <*S*> showed a high level (<*S*> ≈ 0.6) at *E* ≈ 11 kPa, which agrees well with the previous account that reported the optimal striation of actomyosin complexes at *E* ≈ 12 kPa^9^. As shown in Fig. 5f, <S> started decreasing at a much later time point than the shape adaptation as previously reported^64^. In fact, the actin filaments first got shorter before changing their orientation from nematic to isotropic, corresponding the distinct delay before <*S*> started decaying. This finding seems to agree well with the previous accounts, reporting the more pronounced alignment of actin fibers along the major axis according to the increase in substrate elasticity, because the alignment of actin cytoskeletons and the formation of stress fibers are regulated by the force transmission via actomyosin complexes^30,49,65^.

However, in contrast to our previous study using pH responsive gels functionalized with physisorbed fibronectin^29^, we observed no discontinuous change in the order parameter (referred to the break of symmetry) during the mechano-response of C2C12. The different observations might be explained potentially by the following several reasons. First, the characteristic time for the elasticity change caused by the competitive host-guest interaction in this system is much longer than that of hydrogels that change the elasticity by pH change, following a clear difference in the diffusibility of βCD vs. H^+^. In case of “softening”, the change in Young’s modulus completed after 10–30 min (Fig. 2f). This partially overlaps with the time window of cellular response (the lag time ∆*t* ≈ 10 min, the time to reach the equilibrium ≈ 40 min). This might cause a more progressive change in the cytoskeletal ordering. Another scenario is the magnitude of the change in substrate elasticity. In our previous system, the substrate elasticity was modulated from 40 kPa to 2 kPa, which covers a critical substrate elasticity proposed recently by Yip *et al*.; 20 kPa^66^. In this account, it has been suggested that mouse embryonic fibroblast remodels the actin cytoskeletons and retains the substrate strain at a constant level when the Young’s modulus of the substrate was below 20 kPa. On the other hand, they observed the conservation of stress when the Young’s modulus was above 20 kPa, which agrees well with the theoretical prediction^49,67^. Although these studies dealt with different cells (fibroblast and mesencymal stem cells), it is plausible that the critical substrate elasticity at which C2C12 myoblast switches the mechano-response may exist around this level. Actually, the conduction of cardiomyocyte tissues was significantly promoted near 13 kPa^62^. Further studies with substrates with higher elastic modulus (e.g. ≈50 kPa, Fig. 2b) would enable us to determine the critical elasticity jump that causes the symmetry break of the nematic order parameter of actin filaments.

**Figure 6:**
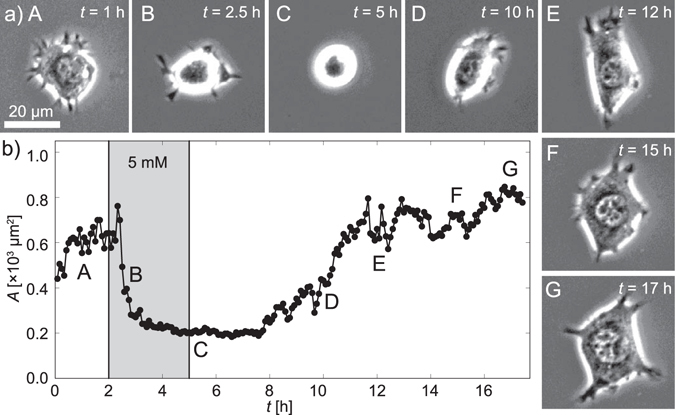
Reversible switching of cell morphology by mechanical commands. (**a**) Some representative phase contrast images are presented in subpanels A – G. C2C12 cells were exposed to medium containing 5 mM of βCD from *t* = 2 to 5 h (shaded time zone). (**b**) Projected area *A* of C2C12 plotted over time. The response of C2C12 to the substrate “softening” from *E* ≈ 11 to 6 kPa occurred much faster than the reverse reaction. The lag time before the cell started responding was also found to be much shorter for the softening. (See Supporting Information Fig. S2 for other examples.)

Figure 6a represents a series of phase contrast images of C2C12 that underwent the reversible switching of its morphology under a medium exchange cycle. Initially, cells were seeded in βCD-free medium. At *t* = 2 h the medium was exchanged to the one containing 5 mM of βCD, and the cell was kept under this condition for 3 h. After confirming the equilibration of the system, the medium was exchanged back to the original βCD-free medium. The projected area *A* of C2C12 was plotted as a function of time in Fig. 6b. The exchange from βCD-free to βCD-containing medium (corresponding to *E* from 11 kPa to 6 kPa) led to a monotonic decrease in *A* after a short lag time (∆*t* = 10–20 min), which is comparable to that presented in Fig. 5. The saturation level of projected area (*A* ≈ 200 µm^2^) seems to agree well with the one obtained from the *ex situ* experiments, *A*_*ex situ*_ = 240 ± 60 µm^2^ (Fig. 4c), confirming that the system reached to equilibrium. At *t* = 5 h, the medium was exchanged back to βCD-free medium. Compared to the “softening” of the host-guest gel substrate, the cellular response to the substrate “stiffening” was found to be much slower. As presented in the figure, the lag time before the visible response was almost 3 h. Such an extremely long lag time here could be explained by the combination of two factors. First, as presented in Figs 2f and 3b, the characteristic time required for the stiffening of host-guest gels is about 1–1.5 h, which is in contrast to the softening that is completed after 10–30 min. Moreover, it is plausible that the spreading of cells requires more time than the contraction. The spreading of cells requires both the establishment of focal contacts and the propagation of spreading fronts. In contrast, the contraction is dominated by cortical tension and the kinetics of actin depolymerization once the focal contacts are cancelled. In fact, the treatment of C2C12 on a stiff substrate (40 kPa) resulted in a fast contraction, while leaving the focal contacts behind^64^. Nevertheless, the projected area *A* increased to 600–800 µm^2^, which is comparable to the initial level (*A* ≈ 600 µm^2^ at *t* = 2 h) and the *ex situ*, equilibrium level, *A*_*ex situ*_ = 620 ± 80 µm^2^ (Fig. 4c). Previously, βCD has been used to deplete the cholesterol and influence the integrin clustering in heat-shocked, Drosophila cells^68^. In this study, we performed control experiments on fibronectin-coated glass substrates (Fig. S2) and polyacrylamide containing no host-guest side chains (Fig. S4), verifying that the presence of βCD does not cause major change in cell shape, adhesion area and cytoskeletal order parameter. Recent studies evidenced that not only the substrate elasticity but also the viscosity influences the dynamic cellular response, such as cell spreading and proliferation^69,70^. The combination with rheological study would enable the optimization of the time windows to investigate the interplay of elasticity and viscosity of substrates.

## Conclusions

In this study, we utilized a new class of hydrogel materials, cross-linked by host-guest interactions, and demonstrated the potential for the dynamic regulation of cell-substrate interactions. Taking the advantage of reversible, supramolecular cross-linkers based on βCD and adamantane, the substrate elasticity can be modulated by exchanging the culture medium with and without free host molecules (βCD). As the modulation of magnitude and direction of the substrate elasticity can be achieved by varying the concentrations of free βCD in solutions, such “host-guest gels” enable one to apply dynamic mechanical stresses to cells by altering the substrate elasticity at a desired time point. In this study, we fabricated a host-guest gel, whose elastic modulus can be adjusted between 4 and 11 kPa. In the first step, we systematically investigated the influence of monomer ratios between host, guest, and matrix monomers on the substrate elasticity. Second, we demonstrated that the elasticity of host-guest gels depends on free βCD concentrations. The projected area of C2C12 on substrates exhibited a clear correlation with the substrate elasticity, and the fitting of the experimental results with the empirical Hill equation indicated that the elasticity of our host-guest gel covers the optimal range for regulating myoblasts. Such *ex situ* experiments under static conditions were further extended to monitor the dynamic, *in situ* response of cells to the change in substrate elasticity. In addition to the commonly used morphological parameters such as aspect ratio and circularity, we tracked the dynamic remodeling of cytoskeletons by calculating the nematic order parameter <*S*> of actin cytoskeletons from live cell images. The softening of substrates led to the detachment of focal adhesion contacts, followed by the morphological change from a stretched, contractile shape to a round shape, reflecting the depolymerization and disordering of actin cytoskeletons. Furthermore, we confirmed such a dynamic mechano-response of C2C12 is fully reversible under softening and stiffening of the gel substrate. Supramolecular hydrogels based on host-guest cross-linkers provide with a major advantage over recently reported materials that can either change the substrate elasticity only in a unidirectional manner or utilize more stressful cues for cells, such as temperature and UV light. Therefore, our host-guest gels open a new avenue not only for understanding the fundamental mechanism of dynamic cellular response to matrix remodeling, but also for a potential to mechanically direct the cell fate in *in vitro* culture.

## Electronic supplementary material

Supporting information
