# Author Correction: Dynamic Mechano-Regulation of Myoblast Cells on Supramolecular Hydrogels Cross-Linked by Reversible Host-Guest Interactions

**DOI:** 10.1038/s41598-018-21593-6

**Published:** 2018-03-06

**Authors:** Marcel Hörning, Masaki Nakahata, Philipp Linke, Akihisa Yamamoto, Mariam Veschgini, Stefan Kaufmann, Yoshinori Takashima, Akira Harada, Motomu Tanaka

**Affiliations:** 1 Institute for Integrated Cell-Material Science (WPI iCeMS), Kyoto University, Kyoto, 606-8501 Japan; 2 Project Research Center for Fundamental Sciences, Graduate School of Science, Osaka University, 1-1 Machikaneyama-cho, Toyonaka Osaka, 560-0043 Japan; 3 Physical Chemistry of Biosystems, University of Heidelberg, D69120 Heidelberg, Germany; 4 Department of Macromolecular Science, Graduate School of Science, Osaka University, 1-1 Machikaneyama-cho, Toyonaka Osaka, 560-0043 Japan; 5 Present Address: Institute of Biomaterials and Biomolecular Systems (IBBS), University of Stuttgart, 70569 Stuttgart, Germany; 6 Present Address: Division of Chemical Engineering, Department of Materials Engineering Science, Graduate School of Engineering Science, Osaka University, 1-3 Machikaneyama-cho, Toyonaka Osaka, 560-8531 Japan

Correction to: *Scientific Reports* 10.1038/s41598-017-07934-x, published online 09 August 2017

The original version of this Article contained a typographical error in the spelling of the author Philipp Linke, which was incorrectly given as Philip Linke. This error has now been corrected in the HTML and PDF versions of the Article.

