# Supplementary material for: Dynamic Mechano-Regulation of Myoblast Cells on Supramolecular Hydrogels Cross-Linked by Reversible Host-Guest Interactions
Source: Sci Rep. 2017 Aug 9;7:7660. doi: 10.1038/s41598-017-07934-x (PMC5550483; doi:10.1038/s41598-017-07934-x)
Supplement: Supplementary file 1 — Supporting information [file 41598_2017_7934_MOESM1_ESM.pdf]

Supporting Information

Dynamic Mechano-Regulation of Myoblast Cells on Supramolecular Hydrogels Cross-Linked by Reversible Host-Guest Interactions

Marcel Hörning[1,†,#], Masaki Nakahata[2,‡,#], Philip Linke[3], Akihisa Yamamoto[1], Mariam Veschgini[3], Stefan Kaufmann[3], Yoshinori Takashima[4], Akira Harada[2,*], Motomu Tanaka[1,3,*]

[1] Institute for Integrated Cell-Material Science (WPI iCeMS), Kyoto University, Kyoto 606-8501, Japan

[2] Project Research Center for Fundamental Sciences, Graduate School of Science, Osaka University, 1-1 Machikaneyama-cho, Toyonaka, Osaka 560-0043, Japan

[3] Physical Chemistry of Biosystems, University of Heidelberg, D69120 Heidelberg, Germany

[4] Department of Macromolecular Science, Graduate School of Science, Osaka University, 1-1 Machikaneyama-cho, Toyonaka, Osaka 560-0043, Japan

# Equal Contributions

Present Addresses:

[†]Institute of Biomaterials and Biomolecular Systems (IBBS), University of Stuttgart, 70569 Stuttgart, Germany

[‡]Division of Chemical Engineering, Department of Materials Engineering Science, Graduate School of Engineering Science, Osaka University, 1-3 Machikaneyama-cho, Toyonaka, Osaka 560-8531, Japan

Contents

S1 Surface functionalization of hydrogel substrates with fibronectin 3

S2 Cell viability test 4

S3 Anisotropic swelling of host-guest gels 5

S4 Additional images of cells on dynamic gels 6

S5 Influence of βCD on cell behavior 7

S1 Surface functionalization of hydrogel substrates with fibronectin.

First, the gel was blot dried, and a 400 μL portion of 100 mM Sulfo-SANPAH in 50 mM HEPES (pH 8.5) was applied. The hydrogel was irradiated with UV light (AS ONE, 254 nm) at a distance of 5 cm for 10 min. The photo-activation procedure was repeated after the removal of Sulfo-SANPAH solution, and the sample was repeatedly washed with 50 mM HEPES (pH 8.5), water, and PBS (2.7 mM KCl, 1.5 mM KH_2PO_4, 137 mM NaCl, 8.1 mM Na_2HPO_4, pH 7.4), respectively. Fibronectin from human plasma (10 μg/mL, Sigma) was layered onto the hydrogel, and allowed to react overnight at 37 °C. Prior to the cell seeding, the gel was washed extensively with PBS and RPMI-1640 medium.

S2 Cell viability test.

The influence of βCD on the viability of C2C12 cells was evaluated using a colorimetric WST-1 kit (Roche Diagnostics GmbH; Mannheim, Germany). 2×10^4 cells per well were seeded into a 96 well plate in RPMI-1640 medium containing 10 % FBS and βCD at concentrations ranging from 0.156 to 10 mM. After incubation for 2 and 4 h at 37 °C under 5 % CO_2, the medium was exchanged to the βCD-free medium containing WST-1 reagent in order to avoid the side reaction between WST-1 reagent and βCD. After incubating the microplate for 2 h, the absorbance of the formazan was measured at a wavelength of 450 nm using a Synergy HTX Multi-Mode Reader (BioTek). The viability was normalized by the signal from C2C12 cells cultured in a βCD-free medium.

Figure S1 represents the normalized viability of C2C12 cells at $t = 2$ h (red) and 4 h (blue). We found no sign of loss of cell viability for all the measured conditions (broken line indicates 100 % level), confirming that βCD does not interfere with the viability of C2C12 cells.

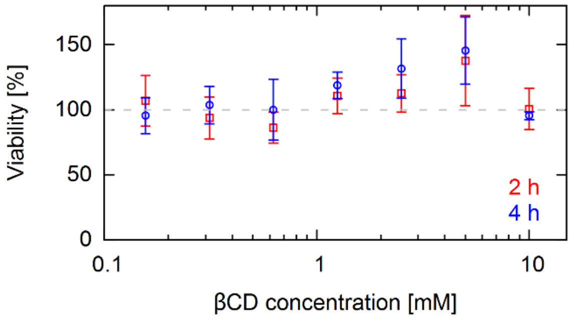

Figure S1. Normalized viability of C2C12 cells incubated in βCD containing medium for 2 h (red) and 4 h (blue). The formazan absorbance from each condition was normalized by that from cells kept in a βCD-free medium. Error bars are calculated from the standard deviation of three independent measurements.

S3 Anisotropic swelling of host-guest gels.

To check if the swelling and shrinking of hydrogels caused by the reversible switching of βCD–Ad bonds in the presence and absence of free βCD molecules in solutions is isotropic, fluorescently labeled latex beads were embedded in the gel. The lateral displacement of beads was tracked with a Nikon C2plus confocal microscope at room temperature using particle image velocimetry (PIV). As presented in Fig. 2c, the lateral bead displacement (≤ 1 μm) is negligibly smaller compared to the change in the thickness measured at the same concentration (≈ 60 μm), implying that the swelling of host-guest gels by free βCD molecules in solution is highly anisotropic.

S4 Additional images of cells on dynamic gels

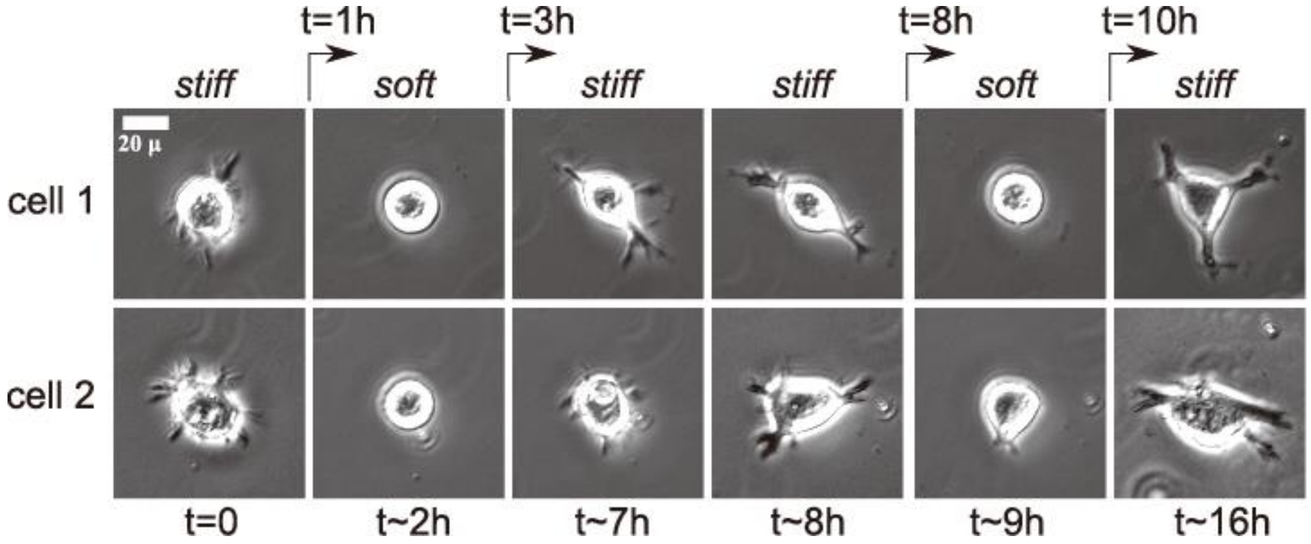

Figure S2. Reversible switching of cell morphology. C2C12 cells were exposed to 5 mM of βCD from t = 1 to 3 h and t = 8 to 10 h. Two cells with representative phase contrast images are presented. The repetitive response of cells to the substrate from $E \approx 11$ to 7 kPa is shown.

S5 Influence of βCD on cell behavior.

To verify the effect of βCD on cell behavior, we cultured C2C12 cells for 24 h on glass substrates pre-treated with fibronectin coated serum. Then, we treat C2C12 cell for 2 h and 4 h with 5 mM βCD (comparable to the condition in Figure 6). The cell spreading, focal adhesion, and cytoskeleton order were monitored by immuno-fluorescence staining of vinculin, actin, and cell nuclei. Moreover, both projected area of cells A and order parameter $<S>$ of actin calculated from $n = 50$ cells also exhibited no significant difference.

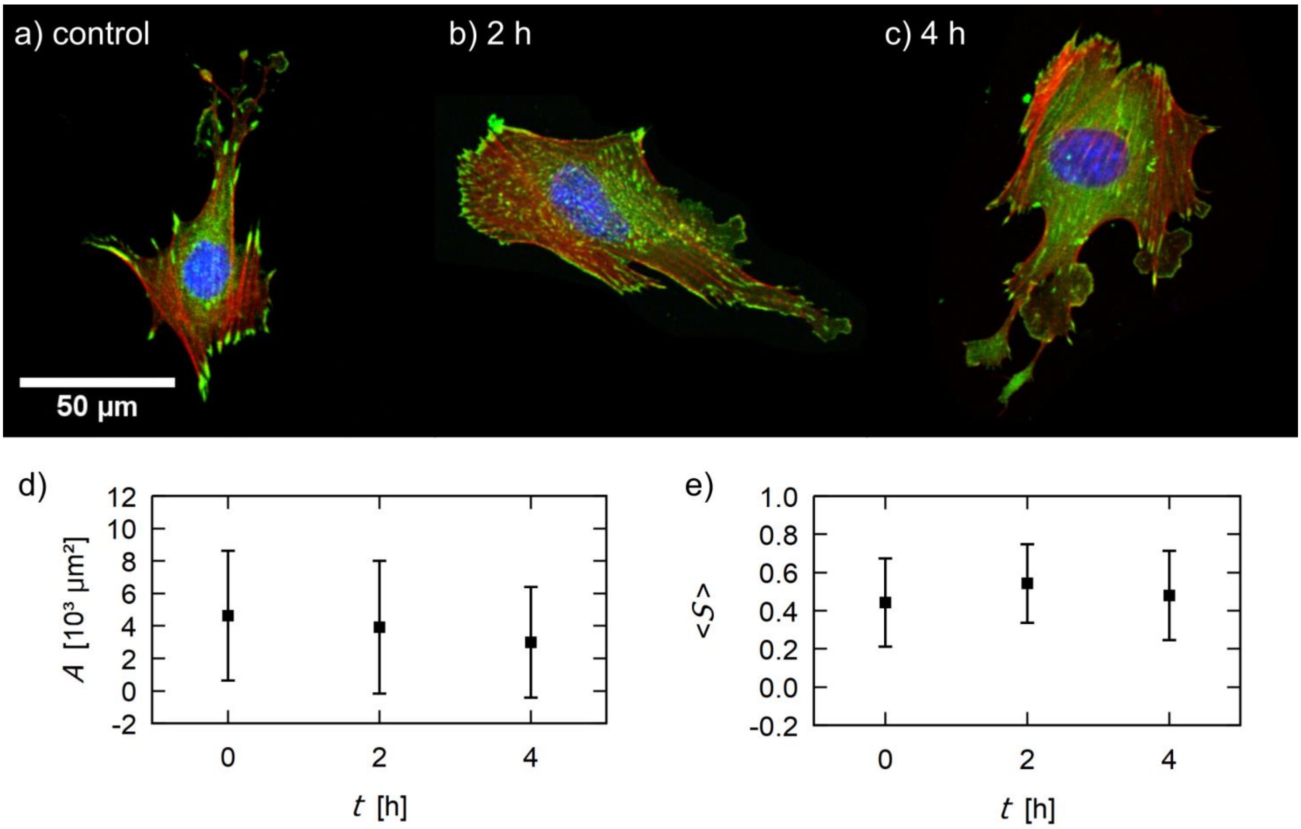

Figure S3. C2C12 cells on fibronectin coated glass (a) before and after treatment with 5 mM βCD for (b) 2 h and (c) 4 h: vinculin (green), actin (red), and nuclei (blue). No significant change in cell morphology was observed. (d) Projected area of cells A and (e) order parameter $\langle S \rangle$ of actin from n = 50 cells for each condition. Error bars show standard deviation.

Along the same line, another series of experiments was performed on chemically cross-linked polyacrylamide gels with no host-guest side chains. Here the Young's modulus was adjusted to be E = 11 kPa. As presented in Fig. S4, the treatment with βCD did not cause any remarkable influence on C2C12.

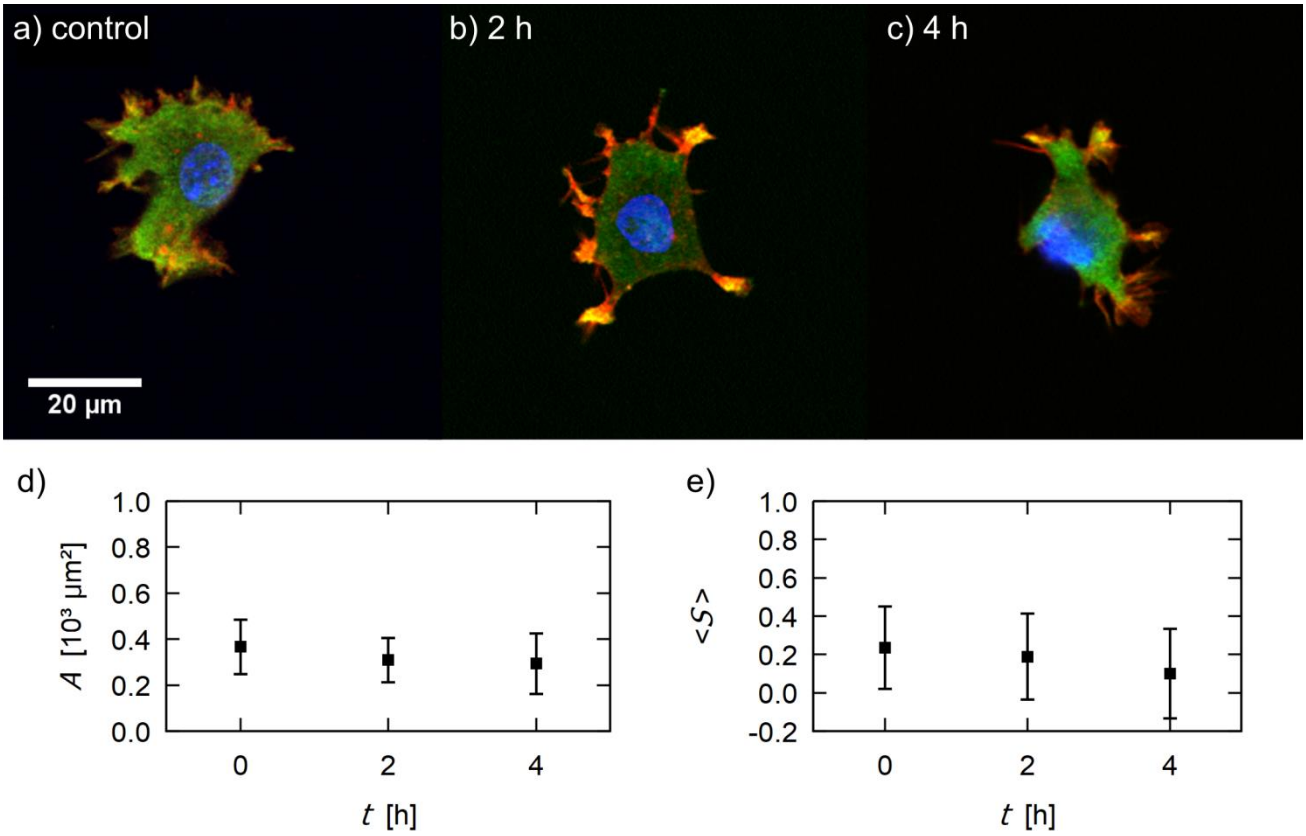

Figure S4. C2C12 cells on fibronectin coated acrylamide gel (a) before and after treatment with 5 mM βCD for (b) 2 h and (c) 4 h: vinculin (green), actin (red), and nuclei (blue). Similar to Fig. S3, no significant change in cell morphology was observed. (d) Projected area of cells A and (e) order parameter $\langle S \rangle$ of actin from $N = 20$ cells for each condition. Error bars show standard deviation.
